# Supplementary figures and images for: Smooth muscle cells affect differential nanoparticle accumulation in disturbed blood flow-induced murine atherosclerosis
Source: PLoS One. 2021 Dec 9;16(12):e0260606. doi: 10.1371/journal.pone.0260606 (PMC8659666; doi:10.1371/journal.pone.0260606)

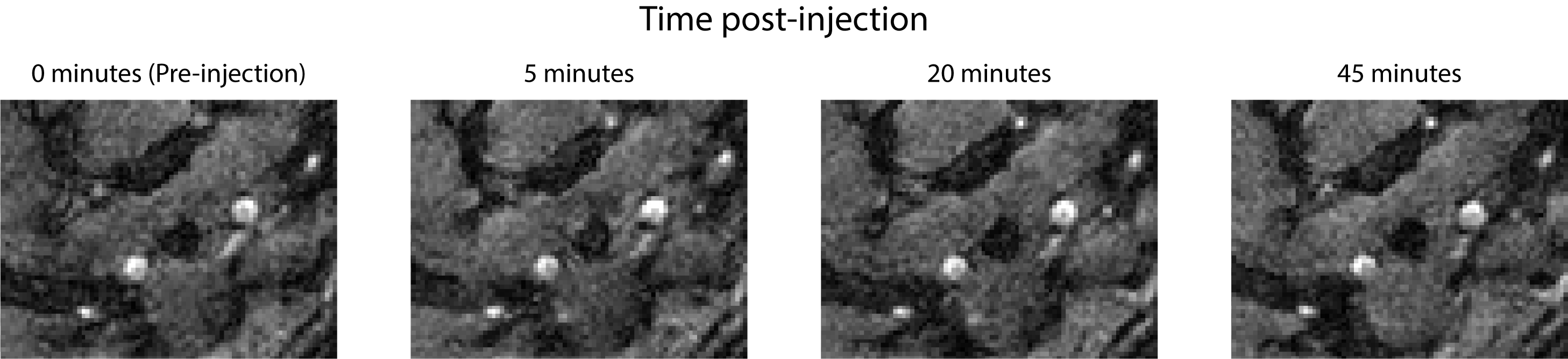

Supplement: S1 Fig — (TIF) [file pone.0260606.s001.tif]

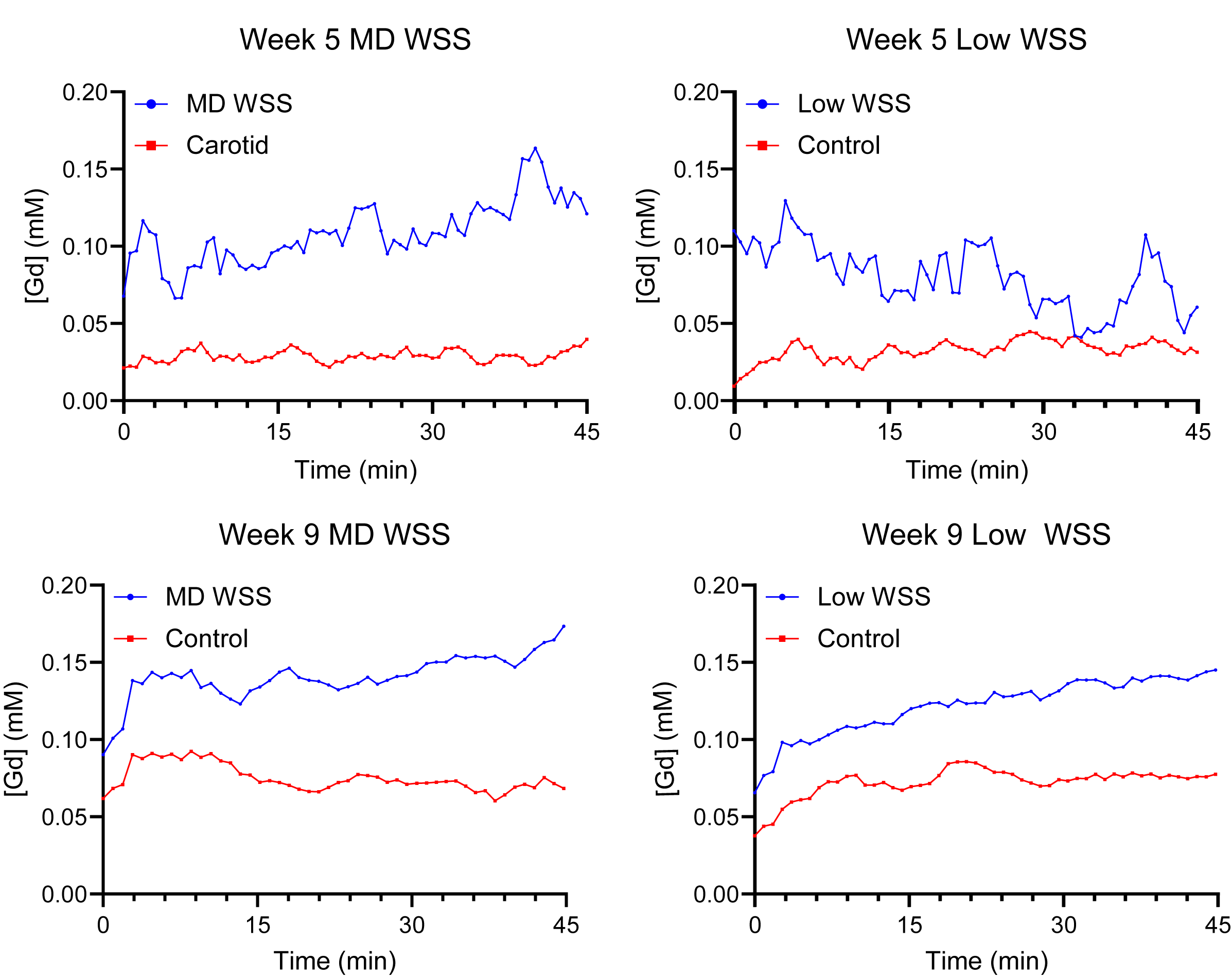

Supplement: S2 Fig — Curves represent a running average of 5 time points taken from a single slice in the relevant vessel region from a single animal. (TIF) [file pone.0260606.s002.tif]

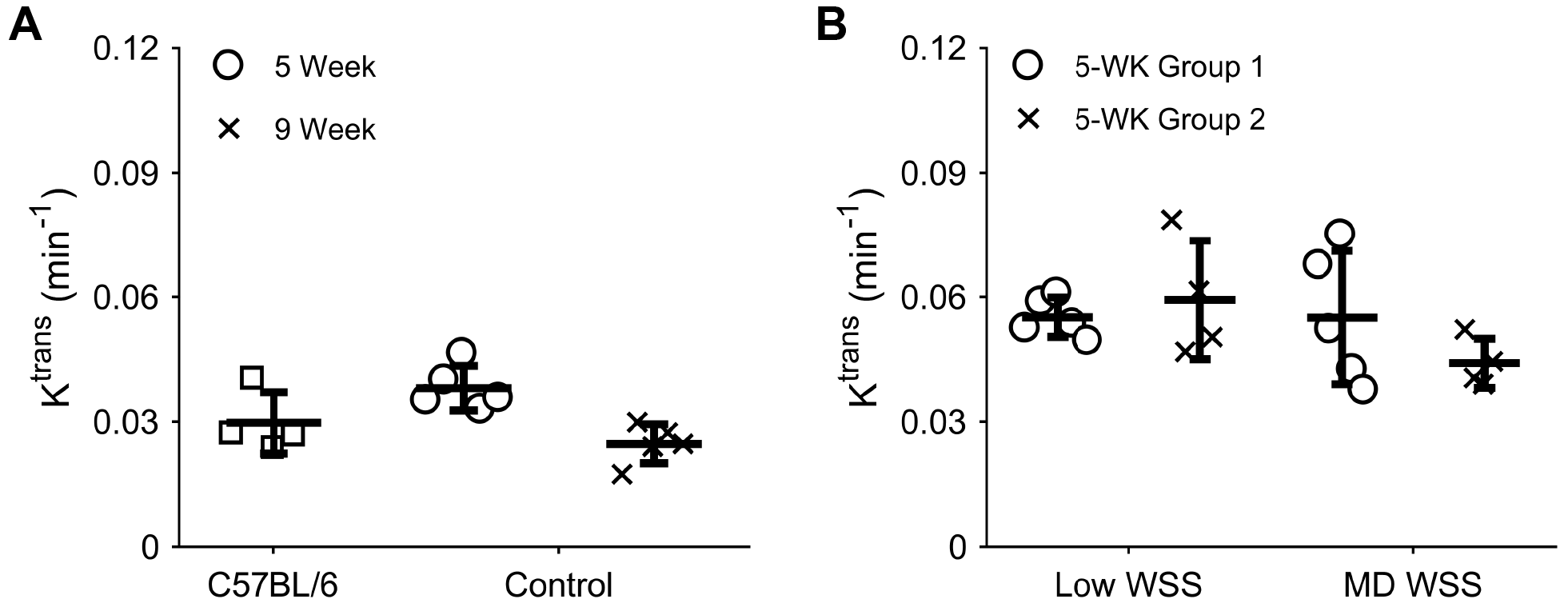

Supplement: S3 Fig — (A) No statistical differences were seen in Ktrans from C57BL/6 mice (four vessels from n = 2 mice) versus control arteries from ApoE mice (n = 5 mice) at 5 and 9 weeks. (B) Four additional mice used for histological evaluation of plaque features at 5 weeks after cuff placement were also injected with NPs immediately prior to culling. No statistical differences in Ktrans were seen in this group of mice (Group 2) versus the five mice injected with NPs at 5 and 9 weeks after cuff placement (Group 1). Each data point represents the mean Ktrans from the central portion of the control artery or the three DCE-MRI slices closest to the cuff within each WSS region of the instrumented artery for each mouse that was injected with NPs. Bars are mean±SD. (TIF) [file pone.0260606.s003.tif]

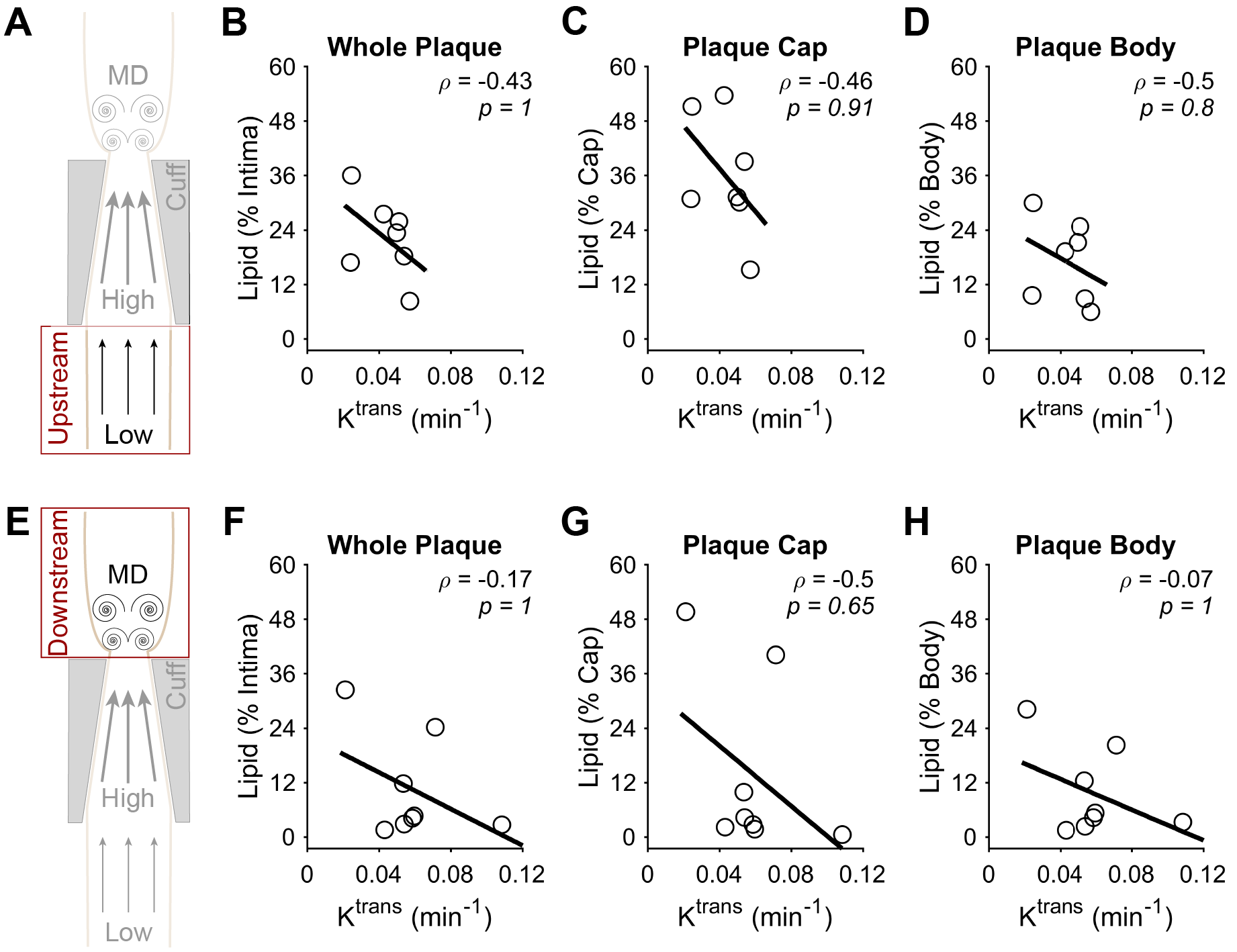

Supplement: S4 Fig — (A) Diagram of the instrumented carotid artery highlighting the focus of this part of the figure on the low WSS (upstream) region. (B-D) The correlation between lipid and Ktrans in different regions of plaques induced by low WSS, including the (B) entire plaque, (C) plaque cap (13.3 μm), and (D) plaque body minus the cap. (E) Diagram of the instrumented carotid artery highlighting the focus of this part of the figure on the multidirectional (MD) WSS (downstream) region. (F-H) The correlation between lipid and Ktrans in different regions of plaques induced by multidirectional WSS, including the (F) entire plaque, (G) plaque cap (13.3 μm), and (H) plaque body minus the cap. Each data point of each plot represents the mean lipid (normalized by intima area) across all histological sections associated with a DCE-MRI slice, from which Ktrans was obtained (in mice with viable histological sections associated with more than one DCE-MRI slice, more than one pairing was used; all mice injected with NPs (n = 5) are represented in all plots). The black line in each plot represents a linear regression of the data to visualize the trend. Spearman’s correlation coefficient, ρ, and associated p-value are also given. *P<0.05 is considered statistically significant. (TIF) [file pone.0260606.s004.tif]

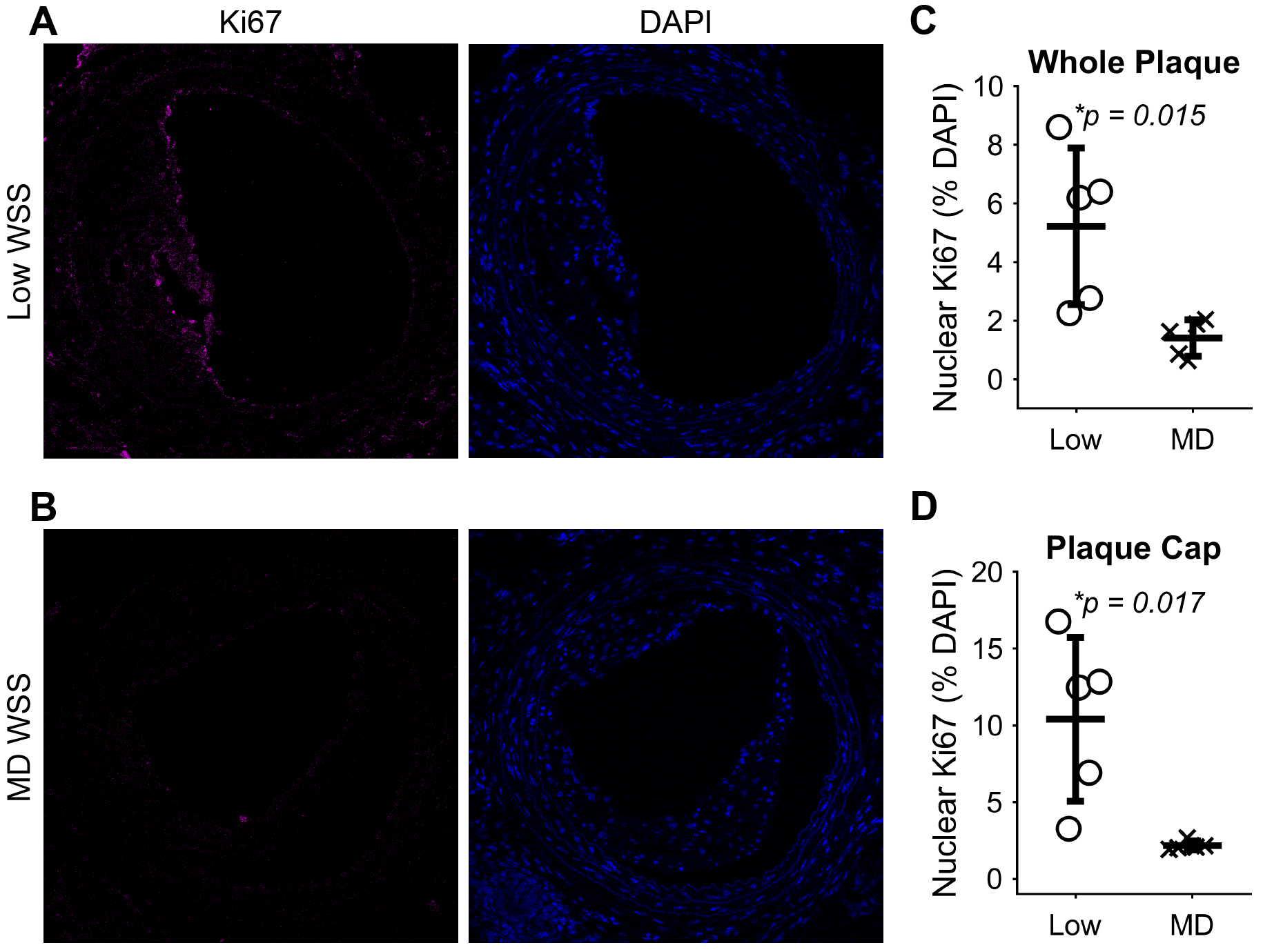

Supplement: S5 Fig — Representative histology sections of Ki67 counterstained with DAPI in plaques induced by (A) low and (B) multidirectional WSS. Plots of nuclear Ki67 area as a percentage of total DAPI area in the (C) whole plaque and (D) plaque cap across all mice evaluated (n = 5). Each data point of each plot represents the average nuclear Ki67 (% DAPI) across all viable histological sections in each vessel segment of one mouse. Bars are mean±SD. *P<0.05 is considered statistically significant. (TIF) [file pone.0260606.s005.tif]
